# Gas Chromatography-Triple Quadrupole Mass Spectrometry Analysis and Vasorelaxant Effect of Essential Oil from* Protium heptaphyllum* (Aubl.) March.

**DOI:** 10.1155/2017/1928171

**Published:** 2017-08-30

**Authors:** Mitra Mobin, Sidney G. de Lima, Lorena T. G. Almeida, José C. Silva Filho, Márcio S. Rocha, Aldeídia P. Oliveira, Marcelo B. Mendes, Fernando A. A. Carvalho, Marcia S. C. Melhem, José G. M. Costa

**Affiliations:** ^1^Centro Universitário UNINOVAFAPI, 64073-505 Teresina, PI, Brazil; ^2^Centro de Ciências da Natureza, Programa de Pós-Graduação em Química, Universidade Federal do Piauí (UFPI), Campus Ministro Petrônio Portella, 64049-550 Teresina, PI, Brazil; ^3^Centro de Ciências da Saúde, Núcleo de Pesquisas em Plantas Medicinais, Campus Ministro Petrônio Portella, 64049-550 Teresina, PI, Brazil; ^4^Centro de Ciências Agrárias, Programa de Pós-Graduação em Ciência Animal, Universidade Federal do Piauí (UFPI), Campus Ministro Petrônio Portella, 64049-550 Teresina, PI, Brazil; ^5^Programa de Pós-Graduação da Coordenadoria de Controle de Doenças, Secretaria de Estado da Saúde de São Paulo, 05403-000 São Paulo, SP, Brazil; ^6^Instituto Adolfo Lutz, 01246-000 São Paulo, SP, Brazil; ^7^Laboratório de Pesquisa de Produtos Naturais, Departamento de Química Biológica, Universidade Regional do Cariri (URCA), 63105-000 Crato, CE, Brazil; ^8^Faculdade de Biomedicina, Centro Universitário Doutor Leão Sampaio (UNILEÃO), 63047-125 Juazeiro do Norte, CE, Brazil

## Abstract

The* Protium heptaphyllum* species, also known as Almécega, produces an oily resin, used in folk medicine as an analgesic and anti-inflammatory agent, in healing, and as an expectorant, which is rich in pentacyclic triterpenes and essential oils. In this study, the essential oil obtained by hydrodistillation of Almécega's resin was analyzed by gas chromatography-triple quadrupole mass spectrometry and evaluated for chemical composition and vasorelaxant activity in rat superior mesenteric artery. The main constituents determined by gas chromatography-triple quadrupole mass spectrometry were limonene, *p*-cineole, and *o*-cymene. In intact rings precontracted with phenylephrine (Phe 1 *μ*M), EOPh (3–750 *μ*g/mL) induced relaxation, and the essential oil had a concentration-dependent vasorelaxant effect, without involvement of endothelial mediators.

## 1. Introduction

According to the World Health Organization (WHO), noncommunicable diseases (NCDs) will be responsible for more than three-quarters of all deaths in 2030, among which cardiovascular diseases (CVD) represent a major risk. The mortality rate by CVD should rise from 17.1 million in 2004 to 23.4 million in 2030. The epidemiological data show that lifestyle and dietary habits are the main factors of the high prevalence of hypertension [1].

Conventional medicines used in the treatment of CVD have numerous collateral effects [2], and they are expensive [3]. Thus, there is a requirement for a safer, cheaper, and more powerful alternative. The professional community has increasingly accepted natural medicine, due to advances in understanding the mechanisms by which plants positively influence health and quality of life [4, 5].

Pharmaceutical studies have shown that natural products represent an extremely valuable source for the production of new chemical products and treatment of untreated diseases [6–8]. Natural products are present in diverse parts of plants, produced through their metabolism, and their pharmacological potential has been confirmed in scientific studies; moreover, they are easily extracted and are economically viable [9]; hence, essential oils have a large tradition in popular medicine [10].

Among the various aromatic plants, present in our region, with powerful pharmaceutical properties, the species* Protium heptaphyllum* (Aubl.) Marchand is highlighted due to its wide distribution in our community, frequently found in core markets, used in folk medicine and extensive potential therapeutic applications.

*P. heptaphyllum*, belonging to the Burseraceae family, is a tree present almost all over Brazil, especially in damp or dry sandy soil areas; it is evergreen or semideciduous and aromatic, with a height of 10 m to 20 m and stem of 40 cm to 60 cm in diameter [11]. It is found mainly in the Amazon region and in some northeastern states, such as Bahia, Ceará, and Piauí, and other countries of South America (Colombia, Paraguay, Suriname, and Venezuela). From its trunk exudes an oily resin called* Almécega* or* Breu-branco*, which hardens in contact with air; this exudate has a greenish-white coloration and a pleasant aroma and is rich in essential oils [11, 12].

Almécega presents several therapeutic indications such as anti-inflammatory, contraceptive, antineoplastic, cicatrizing, expectorant, antimicrobial, and antifungal indications [13]. It is also used by the indigenous Brazilians as a nasal decongestant [10, 14–17] for being present in various parts of the plants produced through its secondary metabolism; its pharmacological potential has been reported in some scientific studies; because they are easy to extract and are economically viable, essential oils have assumed a prominent role in research on natural products; however, still few studies investigate their cardiovascular effects, being the first time reported in the species under study.

The emergence of drug resistant strains of pathogens, the increase in the immunocompromised population, and the limitations of the available antibiotics/drugs have motivated people to use complementary and alternative therapies, including the use of essential oils [18].

The essential oil obtained from the resin of Almécega has been proven to have anti-inflammatory, antitumor, antioxidant, antimicrobial, and cytotoxic actions against* Artemia salina *[15]. In this context,* P. heptaphyllum* has become a potential ally, since many pharmacological studies have shown its positive effects in the treatment of various diseases coupled with the fact that there are no studies that prove the action of this essential oil from* P. heptaphyllum* (EOPh) in contraction of vascular smooth muscle.

## 2. Experimental

### 2.1. Plant Material

Two samples of resins were analyzed in this research, one of which was acquired in the central market of Teresina, PI, in February 2016 (commercial resin) and the other was collected at a private estate in the municipality of Timon, MA, a northeastern Brazilian state, in February 2012 (natural resin) and was kept in refrigeration until the moment of oil extraction. The identification of the species occurred in the Herbarium Graziela Barroso at the Federal University of Piauí, Brazil, and the voucher specimen was registered under number 28730.

### 2.2. Extraction

The resin was subjected to hydrodistillation in a modified Clevenger type apparatus; 3 h after boiling, the oil was collected. The extracted oil was dried with anhydrous sodium sulfate (Na_2_SO_4_), weighed, and stored at a low temperature of 5°C.

### 2.3. Analysis by Gas Chromatography-Mass Spectrometry

The analysis was realized by gas chromatography (Thermo Scientific, TSQ Quantum XLS Ultra, Triple Quadrupole), equipped with an AS3000 Autosampler (Thermo Fisher Scientific Inc., Waltham, MA, USA). For chromatography of the components, a column EquityTM-1, length 30 m, with 0.25 mm internal diameter and 0.10 *μ*m film thickness, was used. Helium was used as a carrier gas with a flow of 1.0 mL/min and injector temperature of 220°C. The column was programmed with an initial temperature of 50°C (3.0 min), followed by an increase of 3°C/min up to 180°C (for 2 min) and then 6°C/min up to 260°C (for 2 min), and detector temperature of 230°C. 1.0 *μ*L was injected into dichloromethane in split mode (1 : 10). The conditions of MS were ion detector triple quadrupole type operating by electronic impact (70 eV; 45 to 450 Da). The identification of the respective components was done by comparing the mass spectra patterns of internal library (NIST 11) to experimental retention index, calculated from an *n*-alkane series (C_8_ to C_20_, Sigma-Aldrich) and by comparison with literal data [19] and also through sites like http://webbook.nist.gov/ and http://www.pherobase.com.

### 2.4. Animals

Male Wistar normotensive rats (200–300 g) were obtained from the Animal Care Facilities of the Center for Research on Medicinal Plants of the Federal University of Piauí, Brazil. The rats were maintained in a large cage under controlled conditions of temperature and lighting (lights on: 06:00–18:00 h). The rats were provided with rodent food and tap water* ad libitum*. All procedures were approved by the ethics committee on animal experimentation of the Federal University of Piauí, Brazil (protocol number 008/12), and were in compliance with the* Guide for the Care and Use of Laboratory Animals* published by the US National Institutes of Health (NIH publication 85-23, revised 1996).

### 2.5. Drugs and Reagents

The drugs used were L-(−)-phenylephrine hydrochloride (Phe), acetylcholine chloride (ACh), verapamil, and cremophor (Sigma-Aldrich, St. Louis, MO, USA). In order to prepare stock solutions of the drugs, all substances were dissolved in distilled water and diluted to the appropriate concentrations. EOPh was dissolved in Tyrode's solution for the* in vitro* protocols using cremophor (0.1% v/v) as the eluent. All solutions were stored at 0°C.

### 2.6. Preparation of the Upper Mesenteric Artery Rings from Rats

The animals were euthanized by thiopental sodium (70 mg/kg i.p.). The superior mesenteric arteries were quickly removed and cleaned of adherent connective tissues and fat. Mesenteric rings (1-2 mm length) were obtained and suspended by cotton threads in an organ bath containing 10 mL of Tyrode's solution, maintained at 37°C, and gassed with a 95% O_2_ + 5% CO_2_ mixture (pH 7.4). The rings were stabilized with a resting tension of 0.75 g for at least 60 min, with replacement of Tyrode's solution (NaCl 158.3 mM, KCl 4.0 mM, CaCl_2_ 2.0 mM, NaHCO_3_ 10.0 mM, C_6_H_12_O_6_ 5.6 mM, MgCl_2_ 1.05 mM, and NaH_2_PO_4_ 0.42 mM) every 15 min to prevent the accumulation of metabolites that could otherwise cause the results to be biased and thus misinterpreted [20]. Isometric tension was recorded by a force-displacement transducer coupled to a data acquisition system (AECAD 1604, AQCAD 2.0.5; AVS Projetos, SP). When necessary, the endothelium was removed by gently rubbing the intimal surface of the vessels with a thin stainless wire and endothelial functionality was assessed through the ability of acetylcholine (10 *μ*M) to induce more than 70% relaxation associated with phenylephrine-Phe (10 *μ*M) tonus. The absence of relaxation following acetylcholine administration was taken as evidence that the rings were functionally denuded of endothelium.

### 2.7. Precontractions Induced by Phenylephrine

After a 60 min stabilization period, a Phe (10 *μ*M)-induced precontraction was elicited in endothelium-intact and endothelium-denuded rings to promote similar magnitude contractions, and EOPh was added cumulatively (3 to 750 *μ*g/mL) after response to Phe had stabilized, approximately 30 min later. In addition, a parallel control was also run under similar experimental conditions with verapamil, a calcium channel antagonist (0.01–3 *μ*M) [21]. Then, a concentration-response curve was obtained.

### 2.8. Data Analysis

All values were expressed as mean ± SEM. The experimental results were expressed as percentage of decrease of the maximum contraction of phenylephrine. The potential was expressed by pD_2_ (anti-log of effective concentration that promotes 50% of maximum response). Student's *t*-test and posttest of Bonferroni were used in the data analysis of the results which were considered significant when *p* < 0.05. All analyses were performed using GraphPad Prism™ software, version 5.0 (GraphPad Software, Inc., San Diego, CA, USA).

## 3. Results and Discussion

**Figure 1:**
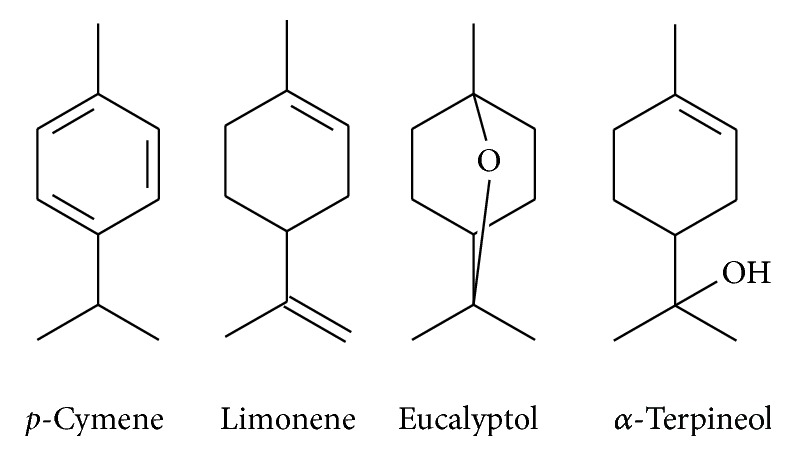
Structures of the main constituents of the essential oil of* Protium heptaphyllum*.

The analysis of the essential oil of* P. heptaphyllum* resin by GC-MS/MS gave rise to the identification of 23 constituents, especially monoterpenes (Table 1). The constituents present in great abundance in the commercial resin were limonene, eucalyptol, and *p*-cymene (Figure 1), whereas in the natural resin, the main components were limonene, *p*-cymene, and *α*-terpineol, with a yield of 1.38% and 0.90%, respectively.

Comparing the EOPh composition with literature data, it was noticed that, generally, phellandrenes, terpinolenes, limonenes, oxidized* p*-menth-3-ene-1,2,8-triol, and aromatized* p*-cymene are major constituents of the essential oil of* P. heptaphyllum*, whereas *α*-pinene was also found in significant amounts of fresh resin oil in most species reported in the literal data. Aged resins of this species, as well as commercial samples (mixture), usually show a lot of phenylpropanoids in their composition. Sesquiterpenes are usually detected in traces in most parts of the reported resin oils, but this ratio was described as an inverse to* P. decandrum* [22].

The chemical constituents of essential oil of resin analyzed by CG-MS are mainly monoterpenes. The oil's chemical composition varies with the period of the year and the region where the material is collected. Citó et al. [23] found that the major compounds were *β*-terpinyl acetate (23.2%), limonene (18.2%), and *o*-cymene (11.2%), while the oil obtained in the municipality of Cruzeiro do Sul, AC, north of Brazil, presented *p*-cymene (39.93%), *n*-tetradecane (13.38%), and dihydro-4-carene (11.69%) as principal constituents [24].

A great amount of oxygenated compounds in commercial resin can be attributed to prolonged exposure to free air in the central market of Teresina, PI; however, we did not notice significant differences in composition, presented in the previous researches.

Recently, Mobin et al. [13] evaluated the chemical composition of* P. heptaphyllum* resins (EOPh) over different extraction times and their antifungal activity against* Candida* species, obtained from gardeners with onychomycosis. Literature review by these authors also showed that the main constituents, mainly monoterpenes, vary significantly according to the extraction period and region where the material was collected.

Literature review showed that the main constituents, mainly monoterpenes, have various pharmacological properties, including antifungal, antibacterial, antioxidant, anticancer, and antispasmodic properties [25–29]. Besides the activities described above, monoterpenes also produce significant effects on the cardiovascular system, promoting, among other actions, vasorelaxation, decreased heart rate, and hypotension [30]. Constituents of the essential oil of* P. heptaphyllum* exhibit biological activities, as* p*-cymene presents anti-inflammatory property [31], D-limonene presents antihyperalgesic action [32], and *α*-terpineol induces gastric protection [33].

In this work, we decided to evaluate the vasorelaxant effect of EOPh for the following reasons: its chemical composition did not change significantly in relation to previous works [13], there is a wide distribution of this species in our region, and it is easy to access the botanical material (resin) throughout the year in the central market of the municipality of Teresina, Piauí state.

**Figure 2:**
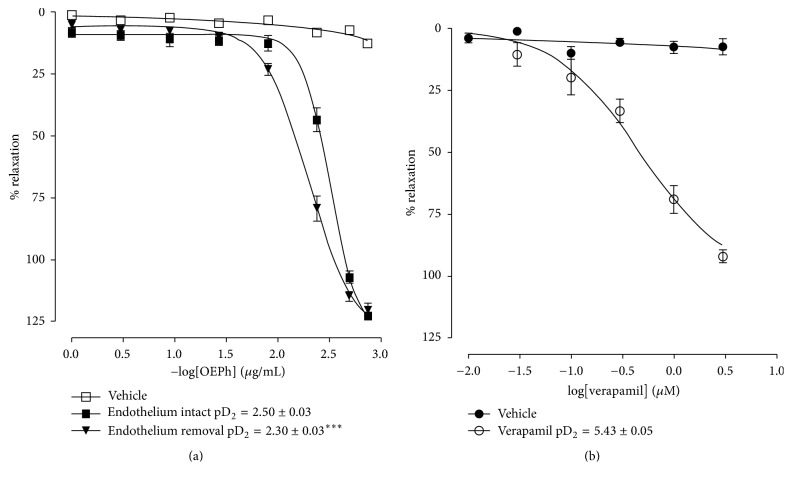
Concentration-response curve showing the vasorelaxant effect of EOPh (a) in endothelium-intact and denuded rat mesenteric rings precontracted with phenylephrine (10 *μ*M) and (b) vasorelaxant effect of verapamil (positive control) in rings of mesenteric artery without endothelium precontracted with phenylephrine. Values are mean ± SEM. ^*∗∗∗*^*p* < 0.001 versus Phe, Student's *t*-test (*n* = 5–7).

In rat mesenteric artery rings, EOPh induced a vasorelaxant response on Phe (10 *μ*mol/L)-induced precontractions. The vasorelaxing effect of EOPh was reduced in preparations in the presence of vascular endothelium (pD_2_ = 2.50 ± 0.03^*∗*^, ^*∗*^*p* < 0.05), in relation to rings without endothelium (pD_2_ = 2.30 ± 0.03) (Figure 2(a)). It is known that Phe-induced contractions are linked not only to Ca^2+^ voltage-sensitive channels but also to receptor-operated Ca^2+^ channels [34]. The vasorelaxant effect of EOPh was similar to the L-type Ca^2+^ channel blocker verapamil, though not with the same pharmacological potency (Figure 2(b)). Several studies have demonstrated that vasorelaxant effect of different plants is usually due to the calcium channel block and hence inhibitors of Ca^2+^ channels are used as antihypertensive agents, such as the ethanol extract of* Mimosa caesalpiifolia* and the essential oil of* Alpinia zerumbet* that induce vasorelaxation by blockade of calcium channels activated voltage, CaV-L [35, 36]. The vasorelaxant effect of EOPh is most evident in preparations without endothelium; this may be due to increased activity of the chemical constituents of the oil on calcium channels in vascular smooth muscle membrane. Therefore, EOPh may have more than one active secondary metabolite leading to a variation in pharmacological response.

Of the 23 monoterpenes identified in this species, five of them had already been studied for their effects on the cardiovascular system:* p*-cymene, eucalyptol, linalool, *α*-pinene, limonene, and *α*-terpineol. The main effects observed were hypotension and bradycardia* in vivo* to* p*-cymene [37], negative inotropic effect* in vivo* to eucalyptol [38], cardiovascular system stimulation and depression* in vivo* to linalool [39], reduction and prevention of cardiovascular injuries caused by pulmonary hypertension* in vivo* to limonene [40], and hypotension and vasorelaxation [41] and antihypertension to terpineol [42]. This paper showed that essential oils may be considered promising agents for prevention or treatment of diseases of the cardiovascular system.

## 4. Conclusion

The analysis of the* P. heptaphyllum* essential oil by GC-MS led to the identification of the major constituents in both commercial and natural resins, limonene, eucalyptol, and *p*-cymene, whereas in the natural resin, the main components were limonene, *p*-cymene, and *α*-terpineol, with a yield of 1.38% and 0.90%, respectively.

In the studies carried out with rat's mesenteric artery, the EOPh promoted a vasorelaxing effect independent of vascular endothelium. These effects provided us with expectancy of a therapeutic alternative for individuals with arterial hypertension. However, further study is needed to investigate the vasorelaxant cellular mechanism of EOPh.

Despite its wide distribution in our community, frequently found in core markets and used in folk medicine and extensive potential therapeutic applications, details on seasonal variation of composition, assessment of toxicity and genotoxicity, and the mechanism of action are important points that should be better evaluated. These experimental data suggest a possible use of EOPh for the treatment of preclinical hypertension.

## Figures and Tables

**Table 1:** Chemical composition of resin essential oil of *P. heptaphyllum*, EOPh.

Constituents	IK^*∗*^	Area (%) EOPh Com. resins	Area (%) EOPh Nat. resins
*α*-Thujene	924	0.19	nd
*α*-Pinene	932	0.71	2.89
Sabinene	969	0.48	nd
*β*-Pinene	971	0.29	nd
*α*-Phellandrene	1002	1.42	7.00
3-Carene	1008	5.11	1.26
*p*-Cymene	1019	17.04	26.87
Limonene	1023	34.51	28.88
Eucalyptol	1025	20.64	nd
*β*-Linalool	1095	0.83	nd
Limonene oxide	1132	0.35	nd
*trans*-Pinocarveol	1135	nd	1.00
Camphor	1141	nd	1.60
4-Terpineol	1174	1.12	1.51
*p*-Cymen-8-ol	1179	1.50	3.22
Criptone	1183	0.80	nd
*α*-Terpineol	1186	9.75	18.39
*trans*-Carveol	1215	1.21	0.92
Cuminal	1238	0.40	1.65
Carvone	1239	1.63	1.27
Piperitone	1249	0.73	1.32
Spathulenol	1577	nd	2.2
*β*-Sinensal	1755	1.29	nd

The identification of the constituents was carried out by comparing the retention index (RI) and the pattern of fragmentation of the mass spectra with literature data and with the database of the analysis system. ^*∗*^Kovat's Index theoretical [19]. nd: not detected or low relative abundance.
